# Factors Affecting Intraoperative Blood Transfusion Requirements during Living Donor Liver Transplantation

**DOI:** 10.3390/jcm13195776

**Published:** 2024-09-27

**Authors:** Hakan Kilercik, Sami Akbulut, Ahmed Elsarawy, Sema Aktas, Utku Alkara, Sinasi Sevmis

**Affiliations:** 1 Department of Anesthesiology and Reanimation, Gaziosmanpasa Hospital, Faculty of Medicine, Istanbul Yeni Yuzyil University, 34010 Istanbul, Turkey; hakankilercik@hotmail.com; 2 Department of Surgery, Liver Transplant Institute, Faculty of Medicine, Inonu University, 44280 Istanbul, Turkey; 3 Department of Surgery and Organ Transplantation, Gaziosmanpasa Hospital, Faculty of Medicine, Istanbul Yeni Yuzyil University, 34010 Istanbul, Turkey; amsarawy@gmail.com (A.E.); semaakt@gmail.com (S.A.); ssevmis@yahoo.com (S.S.); 4 Department of Radiology, Gaziosmanpasa Hospital, Faculty of Medicine, Istanbul Yeni Yuzyil University, 34010 Istanbul, Turkey; utkualkara@yahoo.com

**Keywords:** liver transplantation, living donor liver transplantation, blood transfusion, risk factors, survival

## Abstract

**Background**: Intraoperative blood transfusion (IOBT) during liver transplantation (LT) has negative outcomes, and it has been shown that an increasing number of these procedures may no longer require IOBT. Regarding living donor liver transplantation (LDLT), the literature on the pre-transplant predictors of IOBT is quite heterogeneous and deficient. In this study, we reviewed our experience of IOBT among a homogenous cohort of adult right-lobe LDLTs. **Methods**: We conducted a retrospective analysis of prospectively collected data on adult LDLT recipients between January 2018 and October 2023. Two groups were constructed (No-IOBT vs. IOBT) for the exploration of pre- and intraoperative predictors of IOBT using univariate and multivariate analyses. An ROC curve analysis was applied to identify possible cut-offs. The one-year post-LDLT overall survival was compared using the Kaplan–Meier method. A *p*-value < 0.05 was considered statistically significant. **Results**: A total of 219 adult LDLT recipients were enrolled. The No-IOBT (*n* = 56) patients were mostly males (*p* = 0.016), with higher preoperative levels of HGB (*p* < 0.001), fibrinogen (*p* = 0.005), and albumin (*p* = 0.007) and a lower incidence of pre-transplant upper abdominal surgery (*p* = 0.017), portal vein thrombosis (*p* = 0.04), hepatorenal syndrome (*p* = 0.015), and ascites (*p* = 0.02) than the IOBT group (*n* = 163). The No-IOBT group had a shorter anhepatic phase (*p* = 0.002) and received fewer intravenous crystalloids (*p* = 0.001). In the multivariate analysis, the pre-transplant HGB (*p* < 0.001), fibrinogen (*p* < 0.001), and albumin (*p* = 0.04) levels were independent predictors of IOBT, showing the following cut-offs in the ROC curve analysis: HGB ≤ 11.5 (AUC: 0.800, *p* < 0.001), fibrinogen ≤ 125 (AUC: 0.638, *p* = 0.0024), and albumin ≤ 3.6 (AUC: 0.663, *p* = 0.0002). These were significantly associated with the No-IOBT group. The one-year overall survival of the No-IOBT and IOBT groups was 100% and 83%, respectively (*p* = 0.007). **Conclusions**: IOBT during LDLT is associated with inferior outcomes. The increased need of IOBT during LT can be predicted by evaluating serum levels of hemoglobin, albumin and fibrinogen before liver transplantation.

## 1. Introduction

Since Starzl et al. performed the first successful liver transplantation (LT) in 1967 [1], LT has become the gold-standard treatment for many liver diseases, especially chronic liver disease. While deceased donor liver transplantation (DDLT) constitutes a significant part of LT in Western, developed countries, living donor liver transplantation (LDLT) accounts for a significant part of LTs in many Middle Eastern and Asian countries, including Turkey [2].

Considering the liver’s vascular anatomy, the biliary system, and neighboring organ relations, LT is one of the most complex surgical procedures [1,3] and is made even more difficult by the development of collateral vascular structures associated with portal hypertension and the emergence of dense adhesions between the liver and the inferior vena cava in chronic liver disease [4]. These clinical features predispose patients to additional bleeding, requiring more intraoperative blood transfusions (IOBTs) during LT [4].

IOBTs during LT have been previously associated with negative short- and long-term outcomes, calling for thorough reviews in the LT scientific community over the last few years [5,6,7]. However, most of the reports addressing the IOBT dilemma only covered either DDLT [8,9,10] cases, heterogenous groups [11], or LDLT reports on a small sample size [12].

LDLT is predominantly pursued, for different indications, in regions with low rates of cadaveric donation. IOBT in the LDLT setting is quite different due to the latter’s inherent difficulties; namely, short inflow vessels, complex numerous outflow reconstructions, and frequent double biliary anastomoses. Moreover, the progressive extension of the LDLT landscape to encompass patients with portal vein thrombosis, recurrent HCC, or other criteria is increasing the surgical difficulty and, consequently, the possible need for IOBT [13,14,15,16,17].

For IOBT, ≤3 units of packed red blood cells (RBCs) has been recently set as a quality benchmark in LDLT [18]. IOBT can be practiced within a more restrictive approach [19], obviating the need for it in a reasonable percentage of recipients, taking into consideration the burden of blood preparation on the available resources and the resource waste potential [20]. In this study, we explored the pre-transplant predictors of IOBT in a homogenous cohort of adult right-lobe LDLT cases within a single institution.

## 2. Materials and Methods

### 2.1. Study and Parameter Description

We prospectively reviewed the medical records of all patients (*n* = 328) who underwent LT between January 2018 and October 2023 at the Yeniyuzyil University Faculty of Medicine, Gaziosmanpasa Hospital, Istanbul, Turkey. The following patients were excluded: pediatric LT (*n* = 65), DDLT (*n* = 27), LT due to acute fulminant hepatitis (*n* = 14), and liver re-transplantation (*n* = 3). Finally, a total of 219 cases were included in this study. All the patients received a right-lobe liver graft not including the middle hepatic vein, and operating room management of living liver donors and recipients were performed by the same surgical and anesthesiology team. Recipient hepatectomy was performed without total vascular exclusion, and a portosystemic shunt was not adopted in all patients. Portal vein thrombectomy was performed for 34 patients following the eversion technique, while, in 3 patients, thrombectomy was not feasible, accomplishing inflow restoration instead, using an interposition graft. Graft implantation was performed under partial lateral caval clamping. All anterior sectoral veins were reconstructed if the diameter was ≥5 mm, with a remarkable flow on the backtable, using a polyethylene terephthalate (Dacron) graft. For portal flow modulation, splenectomy and splenic artery ligation were performed in seven and four cases, respectively. An institutional IOBT protocol was followed according to the discretion of the anesthesiology team. In all patients, a leucodepleted packed red blood cell (PRBC) unit (~250 cc) was transfused if the hemoglobin (HGB) level dropped below 7 mg/dL. For those patients with renal impairment and cardiac dysfunction, transfusion was adopted if the HGB dropped below 8 mg/dL and 10 mg/dL, respectively. The early extubation approach was adopted in all cases.

The study cohort was divided into two groups—with (IOBT group) and without (No-IOBT group)—that were compared to identify the pre-transplant predictors of IOBT in LDLT. The following pre- and intraoperative variables were compared: demographics, pre-transplant HGB, platelet count (PLT), international normalized ratio (INR), prothrombin time, total bilirubin, fibrinogen, albumin, Child–Pugh score, model for end-stage liver disease-sodium (MELD-Na) score, previous abdominal surgery, pre-transplant portal vein thrombosis (PVT), hepatorenal syndrome (HRS), ejection fraction (EF), ascites (>1 L encountered upon exploration), spleen diameter (cm; radiologically), entity of esophageal varices (endoscopically and radiologically), entity of splenorenal venous shunt (radiologically), pattern of hepatic vein anastomosis, graft recipient weight ratio (GRWR), anhepatic phase, cold ischemia time (CIT; min), warm ischemia time (WIT; min), total blood loss (mL), and volume of intraoperative crystalloid use (mL). Postoperatively, the re-exploration rate and the length of intensive care unit (ICU) and hospital stays were compared as well. The one-year post-LDLT survival outcome was compared between the groups.

### 2.2. Study Protocol and Ethics Committee Approval

This study involving human participants followed the ethical standards of the institutional and national research committee, alongside those of the 1964 Helsinki Declaration and its later amendments or comparable ethical standards. Ethical approval was obtained from the Inonu University Institutional Review Board (IRB) for non-interventional studies (approval no: 2023/5338). The STROBE (strengthening the reporting of observational studies in epidemiology) guidelines were utilized to assess the likelihood of bias and the overall study quality [21].

### 2.3. Post Hoc Power and Effect Size (Cohen’s d) Calculation

The observed power analysis (post hoc = retrospective power) and effect size were calculated to show the appropriateness of the group comparison and whether the study results were affected by the number of patients in the groups. For this purpose, the mean ± standard deviations of the HGB values in the groups were used. As a result of this analysis, Cohen’s d effect size was calculated to be 1.2 and the power was 100%.

### 2.4. Statistical Analysis

A statistical analysis was performed using SPSS version 25.0 (IBM SPSS Statistics, Amarok, NY, USA) and MedCalc Statistical Software version 22.021 (MedCalc Software Ltd., Ostend, Belgium). The quantitative (continuous) variables were expressed as the median [95% confidence interval (CI), lower and upper bound] and compared with the Mann–Whitney U test. The qualitative (categorical) variables were expressed as numbers (*n*) and percentages (%) and were compared using Fischer’s exact test. Univariate and multivariate analyses were performed to identify significant IOBT predictors, while receiver operating characteristic (ROC) analysis was used to identify the optimum cut-off values of some important quantitative variables (HGB, fibrinogen, albumin, anhepatic phase, MELD-Na score, etc.) to obtain an ideal sensitivity and specificity. Variables with a significance of *p* ≤ 0.05 in the univariate analyses were then used in a multivariate analysis via the backward stepwise logistic regression model to look for an independent factor predicting IOBT in LDLT. The Hosmer–Lemeshow test was used for the goodness of fit of the logistic regression models. The overall survival was estimated using the Kaplan–Meier estimate and was compared with the log-rank test. A *p*-value < 0.05 was considered statistically significant.

## 3. Results

### 3.1. General Assessment

A total of 328 adult patients who underwent LDLT were analyzed for this study. After the exclusion criteria were applied, 219 patients who underwent LDLT constituted the final study cohort. The male-to-female ratio was 139/80. The median (95% CI) recipient age and BMI were 53 years (51–56) and 26.7 kg/m^2^ (26.0–27.7), respectively. The LDLT indications were hepatitis B virus infection (HBV; 26%), cryptogenic (18%), nonalcoholic steatohepatitis (NASH; 18%), alcoholic (10%), autoimmune (9.2%), and others (16%). The median (95% CI) MELD-Na score was 14 (14–16). Most cases (67%) were within the Child–Pugh B category. The number (%) of previous upper abdominal surgery, preoperative ascites, preoperative hepatorenal syndrome, and preoperative portal vein thrombosis were 25 (11%), 193 (88%), 44 (20%), and 37 (17%), respectively. The median (95% CI) operation time and blood loss were 6.0 h (6.0–7.0) and 1000 mL (1000–1500), respectively. The median (95% CI) anhepatic phase was 56 min (53–60). The median CIT and WIT were 42 min (41–45) and 40 min (39–42), whereas the median (95% CI) HGB, PLT, fibrinogen, and albumin levels were 10.8 gr/dL (10.1–11.3), 81,000/µL (73,000–91,000), 124 gr/dL (124–132), and 3.2 gr/dL (3.1–3.4), respectively.

### 3.2. Comparison of Patients with and without Intraoperative Blood Transfusion (IOBT)

**Figure 1:**
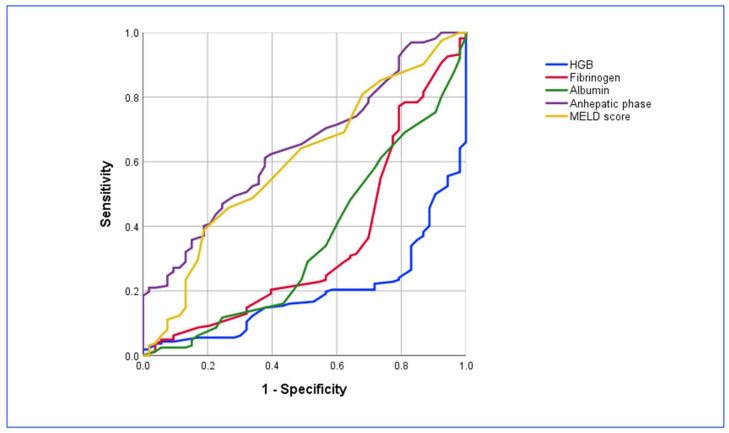
ROC curve analysis of possible cut-offs of pre-transplant predictors of IOBT.

The patients were divided into two groups based on IOBT usage—IOBT (*n* = 163) and No-IOBT (*n* = 56)—where a median (95% CI) of two PRBC units of blood had been given to the former. As shown in Table 1 (preoperative qualitative variables), Table 2 (preoperative quantitative variables), and Table 3 (intraoperative and postoperative quantitative variables), the patients in the No-IOBT group were mostly male (OR: 2.3, 95% CI: 1.2–4.6, *p* = 0.016), with higher preoperative levels of HGB (*p* < 0.001), fibrinogen (*p* = 0.005), and albumin (*p* = 0.007) than the patients with IOBT. Moreover, the No-IOBT patients showed a lesser incidence of pre-transplant upper abdominal surgery (OR: 9.4, 95% CI: 1.3–72, *p* = 0.017), pre-transplant portal vein thrombosis (OR: 3.3, 95% CI: 1.1–9.8, *p* = 0.04), hepatorenal syndrome (OR: 3.9, 95% CI: 1.3–11.5, *p* = 0.015), and intraoperative ascites (OR: 2.9, 95% CI: 1.3–6.7, *p* = 0.02) than their counterparts. Intraoperatively, the No-IOBT group had a shorter anhepatic phase (*p* = 0.002) and received fewer intravenous crystalloids (*p* = 0.001). Postoperatively, the No-IOBT group showed a lower incidence of re-exploration (OR: 3.8, 95% CI: 1.1–13, *p* = 0.02) and stayed for a shorter duration in the hospital than the IOBT cases (*p* = 0.01). In the multivariate analysis (Table 4) to identify the preoperative predictors of IOBT needed during LDLT, HGB ≤ 11.5 gr/dL (OR: 14.05, *p* < 0.001), fibrinogen ≤125 mg/dL (OR: 4.9, *p* < 0.001), and albumin ≤3.6 gr/dL (OR: 2.5, *p* = 0.04) were found to be independent risk factors for the necessity of blood transfusion. An ROC curve analysis was used for the preoperative blood HGB, fibrinogen, and albumin levels and the anhepatic phase (Figure 1).

The ROC curve analyses can be summarized as follows: cut-off values for blood HGB ≤ 11.5 (AUC: 0.800; sensitivity: 73.6%; specificity: 82.1%; 95% CI: 0.74–0.85; *p* < 0.001), albumin ≤3.6 (AUC: 0.663; sensitivity: 83.4%; specificity: 56.4%; 95% CI: 0.60–0.73; *p* = 0.0002), and fibrinogen ≤125 (AUC: 0.638; sensitivity: 68.7%; specificity: 62.5%; 95% CI: 0.57–0.70; *p* = 0.0024) and a cut-off anhepatic phase value >53 min (AUC: 0.651; sensitivity: 61.3%; specificity: 62.5%; 95% CI: 0.58–0.71; *p* = 0.0002).

**Figure 2:**
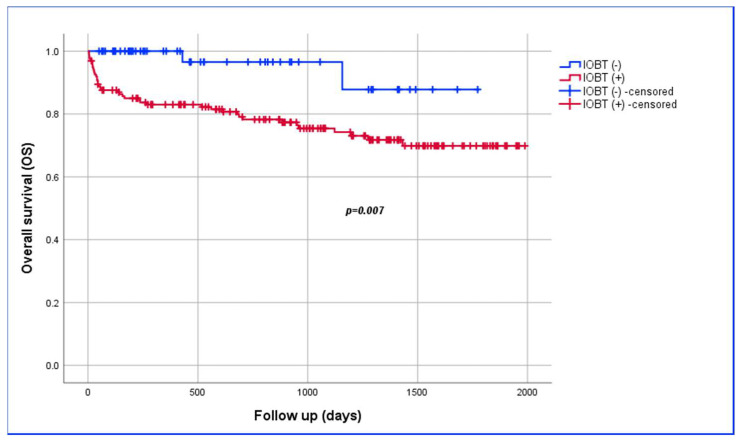
Kaplan–Meier survival estimate of overall survival analysis among patients with or without IOBT.

The percentage of patients who did not receive IOBT progressively increased from 5.5% in 2018 to 55% in 2023. Figure 2 shows the between-group comparison of the one-year overall survival of the No-IOBT and IOBT groups, measuring 100% and 83%, respectively (*p* = 0.007).

## 4. Discussion

In the literature, light has continuously been shed on the issue of IOBT during LT, first and foremost for the purposes of short- and long-term recipient outcomes, as well as quality control, the maintenance of sound surgical practices in this major surgical discipline, and the control of blood banks and other forms of resource exhaustion [22]. LT scientific societies have addressed these points in previous consensus meetings [18], and it has also been recently mentioned that IOBT-free LT is increasingly practiced [23].

LT is recognized as a major surgical procedure, amenable to increasing IOBT demands in view of the blood diatheses relevant to cirrhotic patients’ natural history, graft perfusion-associated hematological changes, and the large surgical site [8]. Nevertheless, a more restrictive IOBT approach is being progressively adopted, though massive hemorrhages are often a possibility.

In our study, a homogenous cohort of adult LDLT patients was reviewed to identify predictors of IOBT need. To ensure the utmost homogeneity of the cohort, pediatric cases, DDLT, LDLT for acute fulminant hepatitis, and liver re-transplantation were excluded. The stability of the surgical and anesthesiology team, cohort homogeneity, and the reasonable sample size added to the robustness of our findings.

Our study showed that 26% of recipients did not receive IOBTs. Previous reports showed an IOBT-free LT range of 10–80%, but most of these were from deceased donors, heterogenous cohorts, or LDLT with a small sample size [10,11,12]. Though a recent report by Singh et al. [24] reviewed a homogenous LDLT cohort, it differs from ours in the fact that the MHV was included in the right-lobe graft. In our patients, the non-inclusion of the MHV increased the surgical complexity/duration and, hence, the possibility of IOBT.

In our multivariate analysis, we found the pre-transplant HGB, albumin, and fibrinogen levels to be significant pre-transplant predictors of IOBT need. Similarly, the preoperative HGB was also found to be an independent predictor of IOBT in previous studies by Massicotte et al. [25], Little et al. [8], and Mangus et al. [26]. Consequently, it was postulated that the preoperative medical treatment of anemia could be associated with decreasing IOBT demands [26,27]. We found that a pre-transplant HGB ≤ 11.5 was associated with an incidence of IOBT nearly 15 times higher than in patients with HGB > 11.5.

Our study also showed that a low baseline fibrinogen level predicts IOBT need, a factor which had been endorsed by previous reports, concurring that fibrinogen monitoring and concentrate use reduce transfusion requirements, making it one the most important indicators guiding intraoperative blood and blood product transfusion [5,23,28]. A recent study by Singh et al. [24] of an LDLT cohort came to the same conclusion using only a univariate analysis [24]. A recent systematic review published by Thibeault et al. [29] showed that high pre-transplant fibrinogen levels were associated with fewer IOBTs, stating that this should be supported by further studies.

Low pre-transplant albumin levels also predicted IOBT necessity in our cohort. McCluskey et al. [11] derived a risk index to predict massive transfusions (≥6 units of PRBCs), with pre-transplant albumin <2.8 g/dL being one of the score elements. Though not statistically significant in multivariate analyses, recent studies by Eghbal et al. [30] and Sobreira Fernandes et al. [31] also identified pre-transplant albumin levels as predictors of IOBT during LT.

In our study cohort, the MELD-Na score (median: 14) was not statistically significantly associated with IOBT. In a study by Massicotte et al. [25], the MELD-Na score was questioned as a valid predictor for guiding IOBT. While the same conclusion was met in a study by Muaddi et al. [10], a report by Sobreira Fernandes et al. [31] with the same median MELD-Na score as our study showed this score to be an independent predictor of IOBT. Again, this conclusion was drawn from a DDLT-only cohort, with the probable presence of confounding factors.

In our study’s univariate analysis, a smaller volume of crystalloids was a consistent finding among cases with No-IOBT. While following a fluid-restrictive approach during LT was associated with less bleeding in previous reports [5,9], it is unclear whether this finding is a cause or a consequence because of the retrospective nature of our study, which required a prospective setting.

The occurrence of previous abdominal surgery constituted a challenging situation in the LDLT context, especially after a previous hepatectomy [17]. In both our patients and those in previous reports [5,23], previous abdominal surgery was a significant factor in preliminary analyses. In a substantial proportion of the literature, IOBT has been associated with reduced graft and patient survival as well as short- and long-term complications [6,26,28]. Indeed, our findings showed a survival of less than one year among those who received IOBT.

The relationship between clinical parameters such as ascites, portal hypertension, and hypersplenism, which are closely related to one another, and IOBT is an important entity requiring investigation. It is a known fact that hepatofugal flow is more pronounced in patients with ascites, making collateral circulation more pronounced and, as a result, making dissection more difficult. Therefore, since surgical bleeding is more common in such patients, more care is necessary. Xia et al. [32] showed a relationship between ascites and IOBT, with Esmat Gamil et al. [33] obtaining similar results. In our study, we found that IOBT need was higher in patients with ascites and PVT, but this significance only remained at the univariate analysis level. Therefore, this is a situation requiring further study support.

The most important direct marker of portal hypertension is the measurement of hepatic venous pressure gradient. However, these measurements are not routinely used during LT. Instead, indirect indicators of portal hypertension such as PLT, spleen diameter, esophageal varices, and splenorenal shunt can be used. In this study, no statistically significant difference was found between the groups when the indicative measurements were examined. However, the relationship between portal hypertension and IOBT can be evaluated by designing prospective studies in which direct measurements will be recorded.

This study has some limitations. Firstly, this study was retrospective, with some independent variables having been incompletely recorded. To overcome this, preoperative, intraoperative, and postoperative patient records need to be standardized, as well as data, which should be recorded prospectively; multicentric studies should be organized in the future, eliminating center-specific effects. Secondly, the groups had low MELD-Na scores (median: 15), indicating that one or more of the patients’ preoperative bilirubin, creatinine, and INR values were low. Although there was no between-group difference in terms of both the MELD-Na score and its two components, the results showed that this score was not effective in these patient groups. In the literature, there are studies showing that the MELD-Na score affects general patient outcomes, as well as others showing that this score has no clinical meaning other than patient allocation for waiting lists. Therefore, we believe that it would be appropriate to conduct prospective and multicenter studies on the relationship between IOBT need and the MELD-Na score. Thirdly, the number of patients in our groups was not compatible, making whether this caused any bias an important issue. The most ideal method for minimizing bias in observational studies, as well as the effect of some independent variables on the dependent one, is using propensity score matching. However, this requires all the independent variables to be complete. To overcome this, we used Cohen’s d effect size, obtained by formulating the mean and standard deviations of continuous variables, showing what the factors indicated in our study. In other words, although the number of patients in the groups was not compatible, we showed that the results were not affected.

## 5. Conclusions

IOBT during LDLT is associated with postoperative complication development and poor patient survival. Predicting IOBT is vital in the pre-transplant period. HGB concentration before the transplant procedure may be a factor that predicts the need for IOBT. The practice of timely pre-transplant blood transfusion to keep the HGB levels ≥11.5 mg/dL would reduce the need for IOBT. Though some authors are questioning preoperative models’ ability to predict IOBT need on a generalizable scale correctly, they might hold valid for local protocols given stable surgical and anesthesiology teams and a steep learning curve.

## Figures and Tables

**Table 1:** Comparison of IOBT and No-IOBT groups in terms of demographic and clinical features (qualitative variables).

Variables	IOBT (*n* = 163)	No-IOBT (*n* = 56)	OR (95%CI)	*p*
Gender (%)			2.3 (1.2–4.6)	0.016
Male	96 (58.9)	43 (76.8)		
Female	67 (41.1)	13 (23.2)		
Child–Pugh Score (%)				0.058
A	14 (8.6)	11 (19.6)		
B	110 (67.5)	36 (64.3)		
C	39 (23.9)	9 (16.1)		
Etiology (%)				0.022
HBV	36 (22.1)	22 (39.3)		
Cryptogenic	34 (20.9)	7 (12.5)		
NASH	34 (20.9)	6 (10.7)		
Alcoholic	13 (8.0)	8 (14.3)		
Autoimmune	12 (7.4)	7 (12.5)		
HCV	5 (3.2)	2 (3.6)		
Others	29 (17.8)	4 (7.1)		
Previous Abdominal Surgery (%)			9.4 (1.3–72)	0.017
Yes	24 (14.7)	1 (1.8)		
No	139 (85.3)	55 (98.2)		
Portal Vein Thrombosis (%)			3.3 (1.1–9.8)	0.040
Yes	33 (20.2)	4 (7.1)		
No	130 (79.8)	53 (92.9)		
HCC (%)				0.140
Yes	32 (19.6)	17 (30.4)		
No	131 (80.4)	39 (69.6)		
Incision Type (%)				0.119
J Type	93 (57.1)	42 (75.0)		
Reverse L Type	38 (23.3)	8 (14.3)		
Chevron Type	16 (9.8)	5 (8.9)		
Reverse T Type	14 (8.6)	1 (1.8)		
Ascites (%)			2.9 (1.3–6.7)	0.020
Yes	149 (91.4)	44 (78.6)		
No	14 (8.6)	12 (21.49		
Hepatic Encephalopathy (%)			3.5 (1.9–6.8)	<0.001
Yes	117 (71.8)	23 (41.8)		
No	46 (28.2)	32 (58.2)		
Hepatorenal Syndrome (%)			3.9 (1.3–11.5)	0.015
Yes	38 (23.5)	4 (7.3)		
No	124 (76.5)	51 (92.7)		
Esophageal varices				0.375
Yes	146 (89.6)	47 (83.9)		
No	17 (10.4)	9(16.1)		
Splenorenal shunt				1.000
Yes	19 (11.7)	6 (10.7)		
No	144 (88.3)	50 (89.3)		
Re-exploration (%)			3.8 (1.1–13.0)	0.040
Yes	29 (17.8)	3 (5.4)		
No	134 (82.2)	53 (94.6)		

HBV: hepatitis B virus; NASH: nonalcoholic steatohepatitis; HCV: hepatitis C virus; HCC: hepatocellular carcinoma; OR: odds ratio: IOBT: intraoperative blood transfusions; 95% confidence interval.

**Table 2:** Comparison of IOBT and No-IOBT groups in terms of demographic and preoperative features (quantitative variables).

Variables [Median (95% CI)]	IOBT (*n* = 163)	No-IOBT (*n* = 56)	Cohen’s d	*p*
Age (years)	54 (53–57)	50 (49–56)	0.09	0.301
BMI (kg/m^2^)	26.7 (26.0–28.0)	26.6 (24.8–28.0)	0.18	0.675
MELD-Na Score	15 (14–17)	14 (13–17)	0.18	0.070
GRWR	1.2 (1.0–1.4)	1.1 (1.0–1.2)	0.03	0.135
Preop HGB	10.0 (9.8–10.8)	12.7 (12.3–13.2)	1.20	<0.001
Preop PLT	83,000 (73,000–96,000)	81,000 (69,000–108,000)	0.10	0.983
Preop INR	1.4 (1.4–1.5)	1.4 (1.3–1.5)	0.24	0.613
Preop Fibrinogen	124 (124–132)	143 (143–154)	0.33	0.001
Preop Creatinine	0.8 (0.7–0.9)	0.8 (0.7–0.9)	0.25	0.161
Preop total bilirubin	1.8 (1.6–2.1)	1.7 (1.2–2.5)	0.07	0.452
Preop Albumin	3.1 (3.1–3.3)	3.6 (3.3–3.8)	0.43	<0.001
Spleen diameter (cm)	16 (16–17)	16 (16–18)	0.07	0.306

BMI: body mass index; MELD-Na: model for end-stage liver disease-sodium; IOBT: intraoperative blood transfusions; GRWR: graft recipient weight ratio; 95% CI: 95% confidence interval; mL: milliliters; ICU: intensive care unit; Intraop: intraoperative; Preop: preoperative.

**Table 3:** Comparison of IOBT and No-IOBT groups in terms of intraoperative and postoperative clinical features (quantitative variables).

Variables [Median (95% CI)]	IOBT (*n* = 163)	No-IOBT (*n* = 56)	Cohen’s d	*p*
Anhepatic phase (min)	59 (55–63)	50 (47–55)	0.62	0.002
Cold ischemia time (min)	42 (40–45)	42 (41–46)	0.08	0.914
Warm ischemia time (min)	41 (38–43)	37 (31–41)	0.17	0.242
Intraop blood loss, mL	1500 (1500–2000)	250 (250–300)	1.63	<0.001
Intraop crystalloids, mL	5600 (5600–6000)	4500 (4000–5360)	0.56	<0.001
Operation time (hour)	6 (6–7)	6 (6–7)	0.33	0.023
Intraop urine output	2000 (2000–2300)	2500 (2400–3000)	0.61	<0.001
ICU stay (days)	1 (1–2) (1.1 ± 0.4)	1 (1–3) (2.0 ± 2.3)	0.55	<0.001
Hospital stay (days)	15 (15–18)	13 (11–15)	0.25	0.014

Cohen’s d (effect size) value was obtained by dividing the mean difference between both groups by the combined standard deviation. Cohen’s d effect sizes are generally classified as small (d = 0.2), medium (d = 0.5), and large (d ≥ 0.8), meaning that, if the difference between two groups is less than 0.2, it is negligible, even if the *p*-value is less than 0.05.

**Table 4:** Multivariate analysis of pre-transplant predictors of IOBT in LDLT.

	B	SE	Wald	df	Sig.	Exp (B)	95% CI	
							Lower	Upper
HGB (≤11.5)	2.643	0.454	33.924	1	0.000	14.056	5.776	34.206
Fibrinogen (≤125)	1.594	0.426	14.005	1	0.000	4.926	2.137	11.354
Albumin (≤3.6)	0.908	0.451	4.056	1	0.044	2.479	1.025	5.996
Anhepatic phase (>53)	0.688	0.407	2.864	1	0.091	1.990	0.897	4.417
Constant	−1.868	0.491	14.497	1	0.000	0.154		

HGB: hemoglobin; 95% CI: 95% confidence interval.

## Data Availability

The data presented in this study are available upon request from the corresponding author. The ethics committee requests a confidentiality letter for the management of this study’s data due to the inclusion of personal and clinical patient data.
